# Authors’ Reply: Beyond Visual Consensus: Tiered Reference Framework for AI Cystoscopy Studies

**DOI:** 10.2196/103335

**Published:** 2026-06-18

**Authors:** Chung-You Tsai, Shi-Wei Huang

**Affiliations:** 1 Division of Urology, Department of Surgery, Far Eastern Memorial Hospital, New Taipei, Taiwan; 2 Department of Electrical Engineering, Yuan Ze University, Taoyuan, Taiwan; 3 Department of Urology, College of Medicine, National Taiwan University, Taipei, Taiwan; 4 Department of Urology, National Taiwan University Hospital, Yunlin Branch, No. 579, Sec. 2, Yunlin Rd., Douliou City, Yunlin, Taiwan, 886 55323911

**Keywords:** multimodal, large language model, AI, cystoscopy, diagnostic reasoning, finding description, biopsy indication, bladder tumor, artificial intelligence

 We thank the reader [1] for their thoughtful and constructive comments on our study [2]. We would like to clarify an important methodological point regarding the reference standard used in our study.

The 401 cystoscopic images were curated from established educational and clinical sources, including reference atlases, PubMed-indexed articles and repositories, clinical websites, industry archives, and Creative Commons–licensed educational videos. These images were originally accompanied by source-provided diagnoses, educational labels, captions, or contextual information.

As stated in the Study Design section, “The reference standard diagnoses were determined through a multiphase, consensus-based process.” To further clarify how source-provided diagnostic information was incorporated, this process can be described in two key phases. In the initial independent review phase, source-provided diagnostic labels were withheld, and two urological experts independently inspected each image and answered the prespecified Q1 to Q5 framework, including anatomic site, cystoscopic findings, lesion detection, lesion reasoning, and final diagnosis. In the subsequent consensus phase, the available source-provided diagnoses or educational labels were disclosed to the experts and considered together with the cystoscopic appearance to resolve discrepancies and establish the final reference answers for Q1 to Q5.

Therefore, the final consensus diagnosis for each image was not based solely on independent visual inspection by two urologists. Rather, the reference standard was established through a source-informed expert consensus process, integrating source-provided diagnostic information with expert review of the cystoscopic appearance.

Thus, the reference standard in our study was designed to support the evaluation of model-generated cystoscopic interpretation and reasoning, rather than to independently re-establish or revalidate the original pathological diagnosis of each image. We agree that histopathology remains the definitive standard for pathological lesion classification, particularly for entities such as carcinoma in situ, papilloma, and papillary urothelial carcinoma. Accordingly, our study should be read as an image interpretation and reasoning benchmark based on images with pre-existing source-provided diagnostic or educational information, with final reference labels established through expert consensus after consideration of both the cystoscopic appearance and the diagnoses provided by the original educational or clinical sources.
